# Embedding research capacity strengthening in multi-country studies in low-and middle-income countries: learnings from sexual and reproductive health research

**DOI:** 10.1080/16549716.2024.2338634

**Published:** 2024-04-12

**Authors:** Anne M. Khisa, Hesborn Wao, Vanessa Brizuela, Rachidatou Compaoré, Adama Baguiya, Alejandra López Gómez, Mercedes Bonet, Seni Kouanda, Anna Thorson, Evelyn Gitau

**Affiliations:** a Capacity Strengthening and SRHR Research, African Population and Health Research Center, Nairobi, Kenya; b UNDP/UNFPA/UNICEF/WHO/World Bank Special Programme of Research, Development and Research Training in Human Reproduction (HRP), Department of Sexual and Reproductive Health and Research, World Health Organization, Geneva, Switzerland; c Department of Biomedical and Public Health, Research Institute for Health Sciences, Ouagadougou, Burkina Faso; d School of Psychology, University of the Republic, Montevideo, Uruguay; e African Institute of Public Health, Ouagadougou, Burkina Faso

**Keywords:** Equity, global health, capacity building, LMIC, sexual and reproductive health

## Abstract

Research capacity strengthening (RCS) can empower individuals, institutions, networks, or countries to define and prioritize problems systematically; develop and scientifically evaluate appropriate solutions; and reinforce or improve capacities to translate knowledge into policy and practice. However, how to embed RCS into multi-country studies focusing on sexual and reproductive health and rights (SRHR) is largely undocumented. We used findings from a qualitative study, from a review of the literature, and from a validation exercise from a panel of experts from research institutions that work on SRHR RCS. We provide a framework for embedded RCS; suggest a set of seven concrete actions that research project planners, designers, implementers, and funders can utilise to guide embedded RCS activities in low- and middle-income countries; and present a practical checklist for planning and assessing embedded RCS in research projects.

## Abstract (plain language summary)

**Paper Context**
- **Main findings:** Building on findings from a primary qualitative study, a literature review, and a consultation with experts on capacity strengthening in LMICs, we propose a systematic approach to embedded RCS.
- **Added knowledge:** We present a framework for embedding RCS in multi-country studies and propose seven action points and a checklist for the implementation of RCS in multi-country research projects with considerations for sexual and reproductive health and rights research.
- **Global health impact for policy and action:** An easy-to-use checklist can enable global health researchers and policymakers to ensure RCS is an integral component of multi-country research.

## Background

Research capacity refers to the ability to conduct high-quality research, disseminate findings and translate knowledge into policy and practice [1]. Research capacity strengthening (RCS) refers to the process of empowering individuals, institutions, networks, or countries to define and prioritize problems systematically. It also includes the process of developing and scientifically evaluating appropriate solutions to health issues and reinforcing or improving capacities to translate knowledge into policy and practice [2]. RCS may include fostering local ownership of research, taking into account the culture and context within which the research is conducted [2,3]. There is a growing number of RCS initiatives at masters, doctoral, and early career researcher fellowships [4–6]. Some of these initiatives are offered by institutions in low- and middle-income countries (LMIC) but financed by high-income countries (HIC) [4–7]. Further, researchers can learn by actively engaging in different stages of research projects through ‘learning by doing.’

Noteworthy, compared to researchers based in HICs, researchers from LMICs tend to have less visibility [8–10], lower publication rates [11,12], and less likelihood of being first or senior authors in publications [12,13]. Some of the factors contributing to this situation include inherent power imbalances, funding inequalities, and language barriers [14–17]. Others argue that RCS problems in LMICs are systemic – involving bureaucratic inefficiency, limited incentive structures for researchers and universities, and limited commitment to research by public and private actors [18]. These concerns resonate with the current calls for *decolonisation* of global health research [19–21]. By decolonizing global health research, we refer to mechanisms to break from existing and persisting power imbalances between funders and implementers as well as between people in different hierarchies and with different roles in the research process.

When RCS efforts are embedded into the full research process, engaging investigators, affected communities, policymakers, and implementers throughout can result in an enhanced sense of ownership, and increased policy-relevance of the research questions being addressed [22]. Embedded RCS can be both a practical and a cost-effective approach to building a pool of researchers who can respond to national and sub-national health priorities. Whereas a number of multi-country embedded implementation research studies have been conducted in LMICs [23–26], only a few have focused on strengthening the capacity of researchers in those countries [27,28]. Furthermore, funders have also focused on embedded RCS efforts. Examples include, among others, the Special Programme for Research and Training in Tropical Diseases (TDR) housed at the World Health Organization (WHO); the United Kingdom Medical Research Council; and the European & Developing Countries Clinical Trials Partnership. Given that the skills developed through embedded RCS present a long-term, sustainable, and equitable approach to conducting research that leads to improved knowledge generation and use, and health system’s performance [29], documenting how to intentionally embed RCS efforts is critical.

Further, evidence on embedded RCS initiatives in multi-country studies focusing on sexual and reproductive health and rights (SRHR) in LMICs is even more scant. Given the sensitive nature of SRHR research [30–32], the capacities of those engaged in it need to be strong, particularly in LMICs, where numerous SRHR studies are conducted [33,34]. Findings from our qualitative study exploring how and whether research capacity was strengthened as a result of participating in the UNDP/UNFPA/UNICEF/WHO/World Bank Special Programme of Research, Development and Research Training in Human Reproduction (HRP)/WHO multi-country global maternal sepsis study [35] suggested that RCS was an unintentional outcome. *Inter alia*, we learned through this study that researchers based in LMICs seek to be well equipped and actively involved in the entire research process instead of ‘just’ playing the role of ‘data collectors’ in studies led by researchers from HICs. While we acknowledge that efforts have been made over the years to overcome some of the issues raised in our study, such as those set forth by the Commission for Research Partnerships with Developing Countries [36], the ESSENCE initiative [37], and until recently by the Council on Health Research for Development [38], challenges remain. Therefore, the objectives of this paper were to provide a framework for embedding RCS in multi-country studies, suggest actions for embedded RCS, and include a checklist to support and monitor the implementation of embedded RCS.

## Methods

We started this exercise using the conceptual framework from our multi-country qualitative study with participants from 16 LMICs [35]. Next, we revised the framework and developed a preliminary list of suggested actions for embedded RCS based on evidence extracted from existing literature on RCS approaches, frameworks, or guidance [3,39–41]. We then conducted a validation exercise of the framework and suggested actions using a panel of experts from research institutions that work with HRP/WHO on SRHR RCS. For this panel, we invited the principal investigators (PI) and co-PIs of seven research institutions located in Brazil, Burkina Faso, Ghana, Kenya, Pakistan, Thailand, and Vietnam, via email. These institutions were selected because they function as regional RCS hubs for the HRP Alliance, a research capacity strengthening initiative from HRP [42]. Each hub provides RCS support to research institutions within their region, responding to pressing SRHR research needs [7]. The validation exercise was held online over Zoom in February 2021 with six PIs/co-PIs, using a question guide to frame the discussion and obtain feedback. Three members of the research team participated in the meeting, convening and moderating the discussion. The panel reviewed the framework and suggested actions, and their recommendations during the meeting together with additional written feedback provided over email were incorporated.

## Findings and discussion

Our final conceptual framework contains five factors for embedding RCS efforts into multi-country studies. We present seven suggested actions for strengthening research capacity, and offer a checklist for planning and evaluating embedded research capacity strengthening in consideration of individual, institutional, and national level actions.

### Conceptual framework for embedding RCS in multi-country research studies

**Figure 1.**
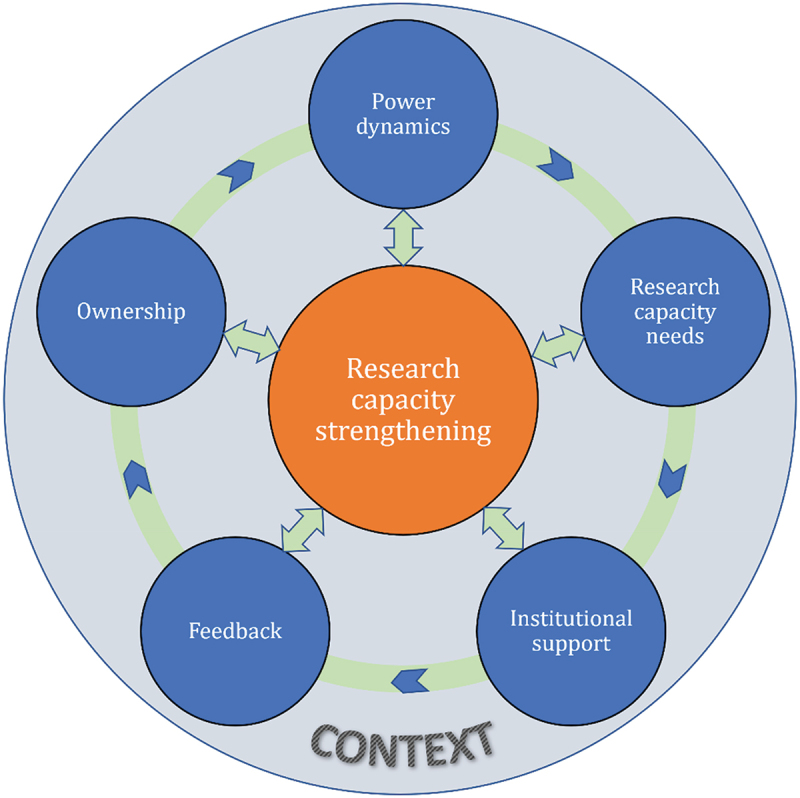
Modified conceptual framework for multi-county embedded RCS initiative [35].

To effectively embed RCS in a multi-country study, we suggest considering the following factors: research capacity needs, institutional support, feedback, ownership, and power dynamics. Each of these concepts is described schematically in Figure 1 and conceptually and through the examples in Table 1.

**Table 1.** Concepts included in the framework for embedded research capacity strengthening.

Concept	Definition
Research capacity needs	Refers to the expressed needs for developing individual knowledge and skills in conducting research and actively engaging in the research process. It includes conducting local research capacity needs assessments and establishing a national research agenda. It helps country teams to develop their priority agenda-setting skills and assess their needs for theoretical and technical knowledge for conducting the research. Specific attention should be made for needs relating to SRHR-related research topics and areas. *As an example, coordinators/conveners of a multi-country study may want to implement a needs assessment survey with all country research teams (including data collectors) to understand potential training needs and knowledge gaps to allow for development of courses or workshops or secure access to training programmes available to all. This should go beyond training specific to the study (i.e. training on protocol implementation).*
Support for researchers	Speaks to the need for having an enabling environment for the implementation of research. These include national and sub-national policies that support research, and especially as these relate to SRHR when applicable. *Oftentimes, study implementers in a multi-country study have to juggle multiple tasks and obligations, including but not limited to clinical practice. Research teams can include funds and time allocation in their study workplans for research-related activities (i.e. data analysis, manuscript writing, dissemination) to ensure researchers have the time and space to conduct all these actions.*
Feedback	Refers to the need for all stakeholders in the research study (e.g. community members participants including women, individuals with diverse sexual orientation, gender identity, gender expression and sex characteristics (SOGIESC), as well as PIs, project managers, data collectors, policymakers) to be included in results dissemination activities in a timely manner and to support sharing of the same to the communities being researched. *Research teams set aside time and funds for dissemination activities that go beyond academic (i.e. through peer-reviewed manuscripts) or institutional circles (i.e. through internal reports) to include the broader community ensuring everyone participating in the study is privy to the findings and can provide input on what is being studied and found.*
Ownership	Relates to the relevance of the study to the researchers’ context and to the fact that their participation in the study design and implementation was deemed critical to the overall execution. This relates to involvement throughout the entire research process from conceptualization of grant proposal or fundraising, planning and designing the study protocol, implementation of study and fieldwork, to data analysis, writing, and dissemination of findings via publication in journals and other outlets. *For example, through open calls for research prioritization and protocol development processes with a mandate to include communities impacted by the study aims, as well as country researchers and policymakers. These would then make up the broader research study team engaged throughout the research process from conceptualization to dissemination of results.*
Power dynamics	This relates to considerations for the potential effect of power differentials (e.g. in funding and roles) that can influence project design and implementation, and in individual RCS. *Among others, one example could focus on ensuring conversations around team composition and roles, and manuscript authorship from the very start that include considerations for equitable representation according to gender, seniority, and affiliation.*

### Suggested actions for strengthening embedded RCS

Several different actions can be taken to improve the embedded RCS in research studies. These actions become critical when dealing with sensitive areas of study, such as those included in SRHR research, which are often politicized and hence deprioritised from national research agendas. Given these contextual factors, we propose actions that can be taken on by different actors at different levels, ensuring that local politics are not limiting areas of study while also laying the ground for research findings to be taken on by national, evidence-based policies. To ensure that RCS efforts are integrated with the research process, short-term RCS opportunities embedded within research can be combined with longer-term, dedicated RCS initiatives. Every stage of the research process (i.e. from conceptualisation, planning, fieldwork, data analysis, writing, and dissemination) offers an opportunity for researchers to be meaningfully involved and empowered. This can be done by ensuring there is a clear understanding of the local context and identification of researchers’ capacity needs prior to planning research studies. Providing researchers with training and a thorough understanding of the whole research process and goal is important for ownership. Having highly skilled researchers able to take on and tackle sensitive research topics and vulnerable populations is integral to improving knowledge on existing SRHR issues.

Despite existing efforts [7, 37, 43], research has shown that much of the health research conducted in LMICs is funded and results published by researchers and partners in HICs without equal involvement or acknowledgement of colleagues in LMICs [44], a reflection of power imbalance and inequality in the research process. For example, oftentimes funders from HICs or institutions located in HICs favour executing agencies located in such settings, rendering eligibility of institutions in LMICs impossible. Furthermore, funders from HIC may support research in LMICs by routing resources through HIC institutions with higher costs and overhead, and who will include their own staffing into projects, thus circumventing and avoiding dealing with institutions based in LMICs directly, further compounding the power asymmetry [15,45]. We suggest incorporating the voices of researchers and communities from the settings where the projects are being implemented to study conceptualization and design, having the implementation process in each country led by national institutions, and full collaboration in the data analysis and writing up of results [46]. Doing this gives researchers based in LMICs equitable opportunity to improve their technical capacity and ability to attract funding, which could provide them with the springboard needed to initiate and lead future multi-country research projects. Shifting power imbalances can also help advance research on different SRHR issues that affect different communities worldwide. Furthermore, we suggest that funders revise requirements that preclude institutions in LMICs from accessing the much needed funds to conduct research locally. For example, to address challenges associated with administration and governance of grant funding by LMIC institutions, funders could require that all institutions seeking funding use the Good Financial Grant Practices, a globally accepted standard for the financial governance and management of grant funding [47].

Design and implementation of large multi-country studies offer opportunities for global health actors to reflect on, and plan for meaningful embedded RCS in health research projects within LMICs. While we advocate for national and subnational empowerment, we also recognize that there may be room and need for institutions located elsewhere, including in HIC, to be engaged in the research process, especially when dealing with issues that counter local practices that curtail individuals’ SRHR (e.g. harmful practices including female genital mutilation and child marriage). International organizations such as WHO should facilitate and be well funded to convene global SRHR partners and ensure that power imbalances are addressed by directly securing research leadership and implementation driven by local actors. This is particularly critical when dealing with SRHR issues that are often understudied at the global level (e.g. abortion, female genital mutilation, and gender-based violence). It is important, however, that if and when multi-country studies are coordinated and/or funded through research institutions based in HICs, these be structured and managed to ensure full equality among all partners in decision-making and data ownership and agency, and credits accrued from undertaking research and disseminating results.

The process of embedding RCS and ensuring research capacity is strengthened, including SRHR research capacity, is determined by multiple factors as delineated above. Aware that there is no one-size-fits-all solution to strengthening local research capacity, we make several suggestions, cognizant of the fact that some of them may be inapplicable in some contexts.

These actions are intended for a wide range of stakeholders entrusted with enhancing equity in health research capacity [48] including community members, funders, donors, civil society organizations, academics, and policy makers. The actions will most likely need to be adapted to fit local contexts and respond to existing needs. Others may be useful as guidance for local, subnational, national, and regional research ethics committees and regulatory bodies in their assessments of research proposals being developed for collaborative implementation in LMICs, especially when these proposals deal with SRHR topics. Our call to action is for all the aforementioned actors because every study offers an opportunity to embed RCS activities and ensure the sustainability of RCS efforts (Table 2).

**Table 2.** Suggested actions for embedding RCS into multi-country research, including sexual and reproductive health and rights (SRHR) research.

1. Involve local researchers and communities throughout all research process, from research question, proposal planning, to protocol development that, in turn, respond to national health needs and priorities.	● Ensure engagement of investigators and the community in all aspects of the research process, aiming for co-creation of research agenda setting, research proposals, grant applications, and protocol development. ● Support local researchers aiming to address critical SRHR topics that may not be reflected in national priorities (e.g. abortion or female genital mutilation). ● Conduct a needs assessment to understand and adapt research projects that respond to the health needs of each context, including on SRHR topics that may not be initially prioritized. ● Advocate for funding agencies to support SRHR research that responds to local needs and not institutional priorities such that the above can be executed.
2. Integrate SRHR research training activities into study design and allocate sufficient resources for it.	● Include RCS objectives into the research protocol to ensure activities are both conducted and reported on. ● Consider attaching local masters or doctoral students in the implementation of the study in their country, to conduct further analyses and lead in publications, as part of their degree programmes. ● Conduct needs assessments prior to research implementation to inform how best to tailor training offerings to bridge identified gaps and include opportunities for learning on intersectional aspects relating to SRHR issues including gender equality and human rights. Training should be made available to all cadres and genders of study teams and on topics covering the entire research process, from conception to dissemination of results, as well as on SRHR subject areas. ● Include financial resources for training and to allow for protected time for those involved in research training when budgeting for study costs. ● Consider online platforms for training activities that are affordable, more time efficient, and can overcome difficulties in travelling. ● Consider involving relevant research study members in the development of training materials and manuals, including leading development of versions in local languages, essential in ensuring SRHR aspects are addressed in a culturally relevant way. ● Plan to measure the effectiveness of the training activities, using rigorous methods depending on appropriateness and need. For example, pre- and post-test surveys can be used to measure short-term impact and satisfaction, while ongoing monitoring – including but not limited to assessing research production from local research teams – can shed light on longer-term and sustainable strengthened capacity. We encourage issuing training certificates to participants who meet pre-specified level of achievement in these activities.
3. Support country-led publication of results in international, peer-reviewed, highly respected journals.	● Ensure authorship is discussed and joint terms of reference for manuscript preparation are formulated before implementing the study, based on guidance provided by the ICMJE [49], while promoting gender equality in authorship and offering opportunities for junior researchers to lead in publications. ● Include financial provisions to support country teams to obtain access to online peer-reviewed journals and for publishing open-access manuscripts to support wider access to knowledge. ● Consider language barriers in both access to publications and writing of results for peer-reviewed journals, promoting diverse manuscript writing groups where identified barriers can be overcome by leveraging group members’ individual capacities. ● Advocate for the removal of publication barriers posed by journals through article processing charges and language requirements. For example, consider abiding by *Coalition S* requirement that scientific publications resulting from research funded by public grants be published as open access publications [50]. ● Request that all publications resulting from the study include authors from the participating countries, including in first and last author positions. No publications should ignore participating country researchers.
4. Support activities to strengthen research infrastructure and provide an enabling SRHR research environment.	● Allocate funding for “protected time” for sexual and reproductive health clinicians (e.g. medical doctors, nurses, midwives) engaging in research studies beyond their clinical responsibilities so that they can truly engage in the research study. ● Support and collaborate with national institutions in fundraising for country led SRHR research studies. ● Include provisions for building the local research infrastructure, such as the capacity of research ethics committees to best respond to SRHR research needs and different research methods, and availability of resources needed for research (e.g. access to peer-reviewed literature, data analysis software). ● Advocate for a more SRHR research enabling environment among funders, parliamentarians, civil society organizations, among others.
5. Address power dynamics in local research teams.	● Ensure local data sets are owned by countries, allowing country study teams to access, manage, and analyse the data as fully as possible. ● Promote the development and strengthening of SRHR networks and exchanges within and across country research teams. ● Ensure gender equality and balance, as well as opportunities for junior researchers, in research leadership and decision-making positions. ● Secure feedback mechanisms throughout project planning and implementation to ensure that researchers are abreast of research progress and results. ● Support the search for national or regional financial sponsorship to break the imbalance between researchers from HIC and LMIC and increase autonomy. ● Include reflexivity statements such as the one suggested by Morton and colleagues [51] that openly address existing power imbalances in research results write-ups.
6. Monitor and evaluate the implementation of embedded RCS.	● Evaluate the RCS interventions for efficacy and impact and develop indicators for measurement of impact. ● Share the lessons learnt and evidence generated with the global communities of practice via various dissemination platforms. ● Design an overarching theory of change for RCS to guide the change process and generate more evidence of change occasioned by the intervention in long-term embedded RCS projects. ● Assess engagement in SRHR research by national institutions following participation in RCS activities.
7. Designate specified funds for RCS support in all SRHR research study budgets.	● Ensure funds are set aside or specific fundraising is initiated to support teams in conducting secondary analyses or ancillary studies that extend beyond the primary data collection phase. ● Facilitate linkages between teaching and research institutions for sustained local skills building in research methodology and analysis, especially for understudied SRHR topics. ● Foster the building of sustainable research networks among country teams to implement subsequent multi-site research. ● Advocate for investments in national health research institutions and researchers as a way to stimulate them as protagonists for gaining scientific independence, especially by governments and national agencies from LMICs.

### Checklist to support implementation and monitoring of suggested actions for embedded RCS

For practical use of these suggestions, we provide a checklist for planning and evaluating embedded RCS interventions in a systematic way. Because RCS may be undertaken at the individual, institutional (organizational), or systemic (national, environmental, or societal) levels [52,53], we propose three levels of RCS interventions, as shown in Table 3. For each level, we suggest actions that the research team should examine to determine whether they are completed where applicable. Of note, some of these actions are easily implemented and monitored in the short term, while others (especially those relating to systemic levels) may need a longer time for implementation and evaluation.

**Table 3.** Checklist for planning and evaluating embedded research capacity strengthening (RCS) in multi-country studies, including sexual and reproductive health and rights (SRHR) studies.

Level	Suggested Action	Completed?	N/A
1. Individual level RCS actions	1. Develop embedded RCS activities that both contribute to agenda setting and the study goal and RCS objective	□	□
	2. Include a training needs assessment for researchers before study initiation	□	□
	3. Tailor activities to meet specific training needs of different cadres of researchers from the data collection team to senior researchers with considerations of SRHR specific issues	□	□
	4. Empower country researchers, including junior researchers and individuals from underrepresented groups as leaders for their teams as appropriate	□	□
	5. Promote gender equality and balance in study research leadership and decision-making positions	□	□
	6. Promote networks and exchanges across country research teams and across multiple countries	□	□
2. Institutional level RCS actions	7. Design project with a theory of change explaining how institutional level RCS will occur	□	□
	8. Evaluate the implementation of RCS interventions and their impact.	□	□
	9. Build the capacity of research ethics committees to assess research protocols and to improve ethics knowledge of research teams	□	□
	10. Assign master’s and PhD students to conduct the analyses and publications in ongoing multi-country studies for sustainable capacity building	□	□
3. National context RCS actions	11. Strengthen national research systems when designing embedded SRHR RCS interventions in LMIC	□	□
	12. Asses the national research capacity strengthening needs and priorities for research including on SRHR critical topics	□	□
	13. Share the lessons learnt with the global community via various dissemination platforms	□	□
	14. Generate and disseminate evidence regarding effectiveness of the planned RCS intervention at national and regional level (including dissemination to civil society organizations)	□	□
	15. Assess uptake of SRHR research by institutions participating in the multi-country studies including embedded RCS activities	□	□

While our call to action and implementation checklist are among many possibilities in accompanying and ensuring equitable, actionable, and sustainable embedded RCS especially as they relate to SRHR research, they can be adapted based on need. Their use and reuse will provide research teams with valuable information on how best to improve this framework and, ultimately, to strengthen the capacity of the research team.

## Conclusion

Participation in research alone is insufficient to sustainably strengthen research capacity. This should be accompanied by a deliberate plan for embedding RCS activities. Embedded RCS can provide the opportunity to address power imbalances in global health research, improve country research capacity in SRHR, increase national research output, and strengthen local research teams to respond to national and sub-national health needs through evidence-informed policies. We recommend actions and provide a checklist as a practical guide for embedding RCS efforts. These actions are therefore an important step in harnessing the potential benefits of embedded RCS in LMICs.
